# Dynamic needle tip positioning versus the angle-distance technique for ultrasound-guided radial artery cannulation in adults: a randomized controlled trial

**DOI:** 10.1186/s12871-020-01152-1

**Published:** 2020-09-14

**Authors:** Bing Bai, Yuan Tian, Yuelun Zhang, Chunhua Yu, Yuguang Huang

**Affiliations:** 1 Department of Anaesthesiology, Peking Union Medical College Hospital, Chinese Academy of Medical Sciences and Peking Union Medical College, Beijing, 100730 China; 2 Medical Research Center, Peking Union Medical College Hospital, Chinese Academy of Medical Sciences and Peking Union Medical College, Beijing, 100730 China

**Keywords:** Catheterization, Radial artery, Ultrasonography

## Abstract

**Background:**

Ultrasound guidance can increase the success rate and reduce the incidence of complications of arterial cannulation. There are few studies on the utility of the dynamic needle tip positioning (DNTP) technique versus the angle-distance (AD) technique for ultrasound-guided radial arterial cannulation in adult surgical patients. We assessed and compared the success rates and incidences of complications of these two short-axis out-of-plane techniques.

**Methods:**

A total of 131 adult surgical patients were randomized into DNTP and AD groups to undergo ultrasound-guided radial artery cannulation. The primary outcome was first-pass success without posterior wall puncture. The secondary outcomes included the first-pass success rate, 10-min overall success rate, cannulation time, posterior wall puncture, and the number of skin punctures.

**Results:**

The first-pass success rates without posterior wall puncture were 53.8% in the DNTP group and 44.6% in the AD group (RR = 1.22, 95% CI: 0.86–1.72; *P* = 0.26). The cannulation time was significantly longer (*P* = 0.01) in the DNTP group [79.65 (54.3–109.4) seconds] than in the AD group [47.6 (24.9–103.8) seconds]. The posterior wall puncture rate was significantly lower (*P* = 0.002) in the DNTP group (29.2%) than in the AD group (56.1%; RR = 0.56, 95% CI: 0.42–0.82).

**Conclusions:**

There were no significant differences in the first-pass success rate, with or without arterial posterior wall puncture, or in the 10-min overall success rate between the DNTP and AD groups. However, the cannulation time was longer and the posterior wall puncture rate was lower in the DNTP group.

**Trial registration:**

The trial was registered at www.clinicaltrials.gov (No: NCT03656978). Registered 4 September 2018.

## Background

Arterial cannulation is a common invasive procedure that enables beat-to-beat blood pressure monitoring, frequent blood sampling and the assessment of fluid responsiveness in operating rooms and intensive care units [1, 2]. Different sites can be chosen for arterial cannulation, but the radial artery is most commonly used due to its superficial course, its dual blood supply to the hand, and a low rate of complications [3]. Complications from arterial cannulation include infection, hematoma, vasospasm and nerve injury [4, 5]. Studies have shown that the success rate is higher and the incidence of related complications lower when performing this technique with ultrasound guidance than when performing it with traditional palpation [6–8].

Using ultrasound, vascular structures can be viewed in two orientations: short-axis/out-of-plane or long-axis/in-plane [9]. The short-axis view has the advantages of providing better visualization of the surrounding structures and easier imaging. The traditional short-axis technique, that is, the angle-distance (AD) method, is accurate in terms of positioning and is convenient for novices to master. Earlier studies have suggested that using the AD technique can significantly improve the success rate of cannulation. However, despite the advantages of the short-axis technique, such as accurate positioning of the puncture point and visualization of the relevant perivascular structures, posterior wall puncture of the target artery might not be avoided [10, 11].

In recent years, studies have reported that a modified ultrasound-guided short-axis technique, i.e., the dynamic needle tip positioning (DNTP) technique, is superior to the palpation technique in both adult and neonate surgical patients [6, 12]. Researchers have found that when using the modified technique, after seeing the blood return, the probe needs to be moved further to ensure that the tube is in the arterial lumen before the next step is performed, and this might reduce the risk of posterior wall penetration [8, 13, 14]. However, this modified method requires frequent movement of the probe, which requires more operator experience and might take more time [15]. Notably, no studies have compared the success and complication rates of the DNTP technique and the AD technique. Therefore, we believe that it is necessary to compare the two techniques. We compared these two techniques and determined the strengths and weaknesses of each to provide a reference for the clinical choice of a specific technique.

## Methods

### Ethics and registration

This trial was approved by the institutional review board for human studies at Peking Union Medical College Hospital, Peking Union Medical College (Institutional Review Board #zs1338), and written informed consent was obtained from all subjects participating in the trial. The trial was registered at www.clinicaltrials.gov prior to patient enrolment (number: NCT03656978; principal investigator: Y.T.; date of registration: September 3, 2018).

### Randomization and blinding

**Fig. 1:**
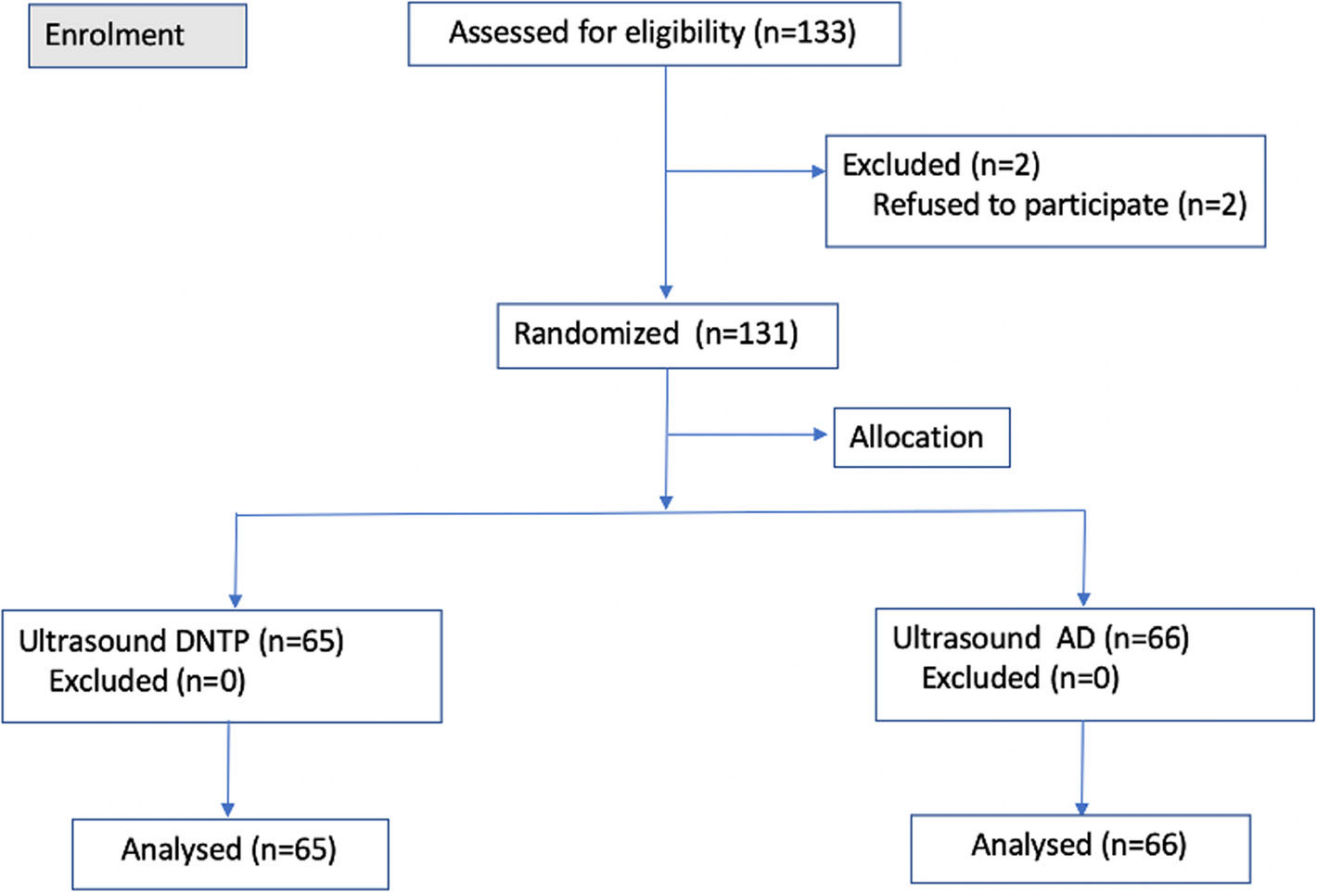
CONSORT flow diagram

The trial adheres the applicable CONSORT guidelines. Patients scheduled to undergo elective surgery and clinically require arterial cannulation at Peking Union Medical College Hospital (Beijing, China) were enrolled from September 2018 to February 2019. The inclusion criteria for this trial were patients undergoing elective surgery clinically requiring arterial cannulation, older than 18 years and with ASA level I-IV. The exclusion criteria were contraindications for peripheral arterial puncture or catheterization, a blocked or embolized target vessel determined by ultrasound assessment, and patient refusal (Fig. 1). The anesthesiologist who conducted the cannulation procedure knew the allocation of the patients. The patients were blinded to the allocation. The statistician did not know the allocation. Enrolled patients were randomized by computer-generated numbers provided in sealed opaque envelopes to either the DNTP or AD group. The seal of the envelope was broken just before the cannulation procedure. The operator was an experienced senior anesthesiology resident in our department, who had already conducted the DNTP and AD techniques in over 100 patients each and was equally skilled in the 2 methods. An intention-to-treat analysis was used in this trial.

### Intervention and control

In the operating room, after securing intravenous access and routine monitoring using electrocardiography, pulse oximetry, and noninvasive blood pressure, patients were given 1 mg of midazolam iv and 50 μg of fentanyl iv as well as oxygen through a mask. The wrist of the patient was extended over a roll to raise the arm and stabilized by taping it to an arm board. An ultrasound machine (Navi s; Wisonic, Inc., Shenzhen, Guangdong, China) and probe (L15-4NB; Wisonic, Inc.) were used before the procedure based on randomization to obtain an image of the radial artery and acquire measurements. Sterile techniques, including sterile gloves, operating towels, and ultrasonic probe covers, were used for arterial catheterization. After sterile skin preparation, ultrasound-guided arterial cannulation was performed using a 20-gauge intravenous catheter (BD Angiocath, Becton Dickinson Infusion Therapy Systems, Inc., Sandy, Utah, USA). The patients were administered 0.2 ml of 2% lidocaine for local anesthesia at the insertion site before the cannulation procedure.

For both techniques, a timer was started when the operator was ready to use the needle to pass through the skin and stopped when an arterial waveform appeared. If the procedure lasted more than 10 min, the operator was forced to stop and free to use any technique until success was achieved. In that case, the cannulation was recorded as 600 s. The research team defined first-pass success as the successful acquisition of a regular waveform on the screen after just one pass through the skin.

**Fig. 2:**
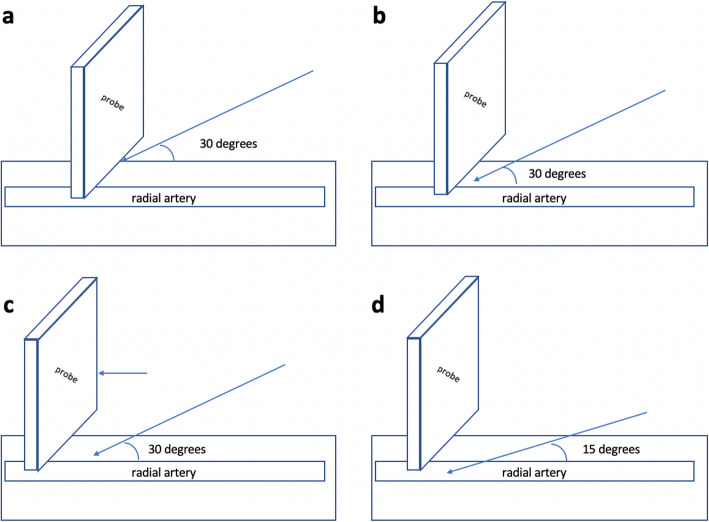
Diagrams showing how to advance the needle into the radial artery using the DNTP technique. **a**, Obtain an image of the target artery and adjust the insertion point and orientation of the needle. **b**, Puncture the target artery, and observe the needle in the artery lumen. **c**, Advance the ultrasound probe away from the needle until the hyperechoic needle is no longer observed. **d**, Advance the needle and catheter until the very tip is seen again, and repeat these steps until the tip is observed in the artery lumen

For the DNTP technique, the probe was placed to view the out-of-plane radial artery and moved to place the artery in the center of the ultrasound screen. Then, the needle was inserted at the point at which the middle mark of the probe contacted the skin and advanced through the skin at an angle of approximately 30 degrees until the tip was seen on the screen (Fig. 2). The probe was moved along the long-axis of the target artery away from the insertion point until the tip just disappeared from the screen. Then, we advanced the needle and catheter until the tip was just seen again. These steps were repeated until the tip was observed in the artery lumen. The cannula was then inserted into the artery, and the needle was removed.

**Fig. 3:**
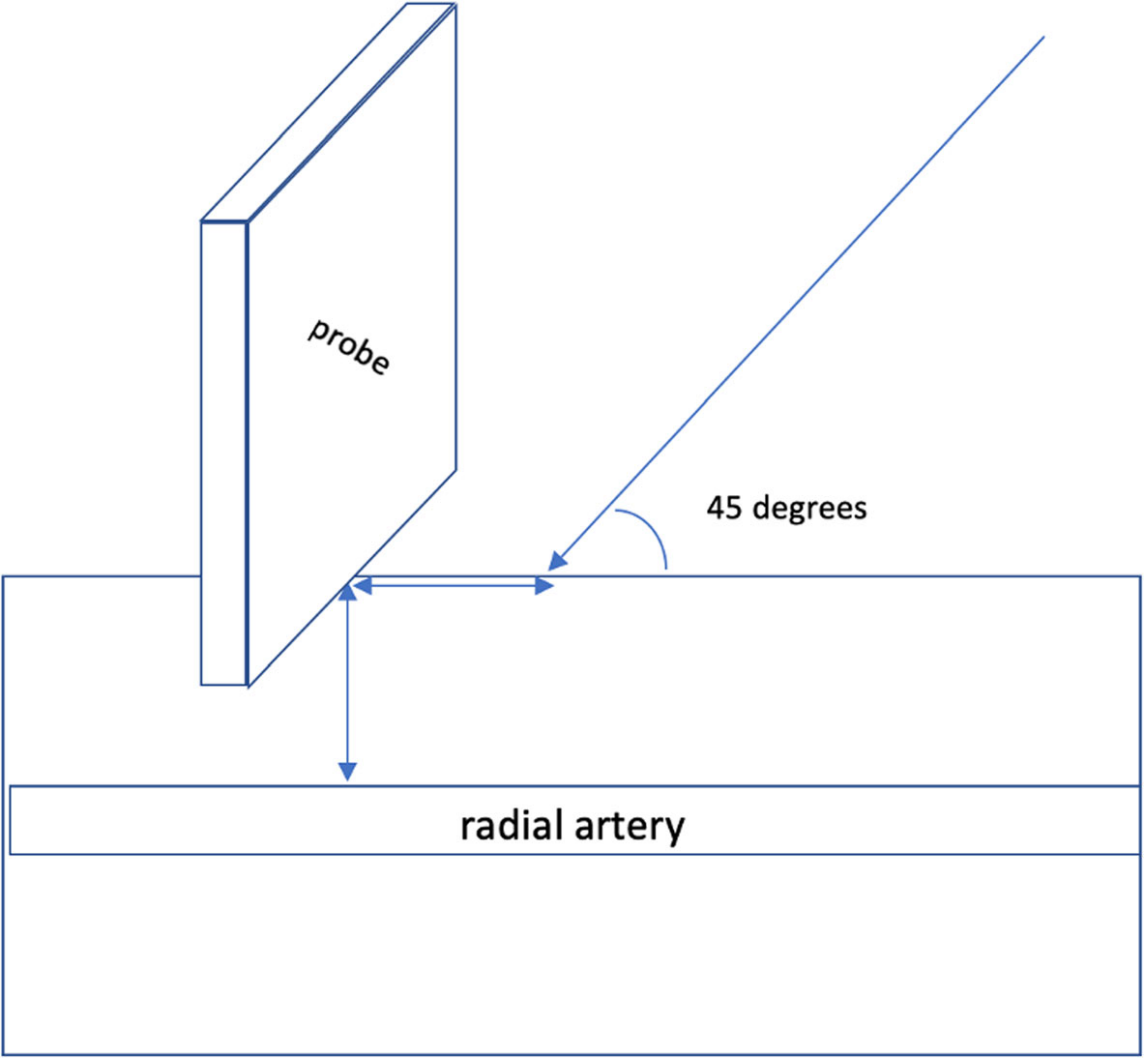
Insertion point and orientation of the needle when using the AD technique

For the AD technique, the probe was placed to view the short-axis plane of the target artery and moved to place the artery in the center of the ultrasound screen. Then, the distance from the surface of the skin to the anterior wall of the artery was measured. The needle was inserted at the point at which the middle mark of the probe contacted the skin and advanced through the skin. Since an initial angle of 45 degrees between the needle and skin was used for puncture, the distance between the point of insertion and the central point of the probe was approximately equal to the distance from the surface of the skin to the anterior wall of the artery (Fig. 3). Then, the needle was advanced until blood appeared in the hub. The needle angle was decreased slightly while the catheter was advanced slightly. The catheter was advanced into the target artery only if blood continued to flow into the hub. If insertion failed or the target was missed, the needle insertion direction was adjusted according to the position of the needle tip.

### Outcomes

The following baseline data were collected for each patient: sex, age, height, weight, body mass index, systolic blood pressure, diastolic blood pressure, heart rate just before the procedure, history of hypertension, diabetes, coronary heart disease or smoking, surgery type, radial artery diameter and radial artery depth. The primary outcome was first-pass success without posterior wall puncture. The secondary outcomes included the first-pass success rate, the 10-min overall success rate, the cannulation time, posterior wall puncture, and the number of skin punctures. First-pass success without posterior wall puncture was defined as successful catheterization without vascular damage to the posterior wall. First-pass success was defined as successful catheterization on the first attempt regardless of vascular damage to the posterior wall. Overall success was defined as successful catheterization regardless of vascular damage to the posterior wall and without a limit on the number of punctures. The cannulation time was defined as the interval between skin contact with the probe and confirmation of the arterial waveform on the monitor. Posterior wall puncture of the radial artery was considered when the operator saw the needle passing the posterior wall or blood backflow appeared then disappeared while needle advancing. The number of skin punctures refers to the number of skin punctures that occurred during the whole cannulation procedure.

### Statistical analysis

The baseline characteristics of the participants are described using descriptive statistics. The clinical and demographic characteristics of patients in the two groups were compared using the t-test, the chi-square test, and nonparametric rank tests, and a *P* value less than 0.05 was considered to indicate a statistically significant imbalance between the groups. When analyzing the primary outcome, imbalanced baseline characteristics were adjusted using logistic regression analysis. The primary outcome, first-pass success without posterior wall puncture, was compared using the chi-square test and relative risk with the corresponding calculated 95% confidence intervals. Similarly, the secondary outcomes, which included first-pass success, 10-min overall success, posterior wall puncture and the number of skin punctures, were also compared using the chi-square test. The median cannulation time was compared using the Mann–Whitney U test. Statistical analysis was conducted on an intention-to-treat basis using SPSS (version 25.0; IBM Corp). A two-sided *P* value less than 0.05 was considered statistically significant.

### Sample size calculation

In our preliminary trial, the first-pass success rate was 77% with the DNTP technique and 53% with the AD technique. To compare the 53% first-attempt success rate in the AD group with the 77% rate in the DNTP group, with a power of 80% and a two-sided type I error rate of 0.05, we needed to enroll 61 patients per group. Considering a dropout rate of 10%, a total of 136 patients were planned for enrolment.

## Results

A total of 136 patients were recruited and screened for eligibility in the trial from September 2018 to February 2019. Five of the patients were excluded from the trial because they refused to sign the consent form. Finally, we randomized 131 patients who underwent a nonemergent operation and required radial arterial cannulation in the final analysis, as shown in Fig. 1. There were 79 (60.3%) males and 52 (39.7%) females, and the average age was 58.27 ± 13.26 years. There were 65 patients in the DNTP group and 66 patients in the AD group. The basic characteristics are shown in Table 1, and there were no significant differences between the two groups.

**Table 1:** Baseline characteristics of all patients enrolled in the trial

	Technique		***P*** Value
	DNTP (***n*** = 65)	AD (***n*** = 66)	
Male, n (%)	39 (60.0)	40 (66.6)	0.94
Age, y	59 ± 14	58 ± 13	0.68
Height, cm	166 ± 8	169 ± 8	0.11
Weight, kg	68 ± 9	70 ± 6	0.16
Body mass index, kg/m^2^	25 ± 3	25 ± 3	0.83
ASA level			0.80
I-II	17	16	
III-IV	48	50	
Systolic blood pressure, mmHg	143 ± 20	149 ± 19	0.09
Diastolic blood pressure, mmHg	74 ± 21	79 ± 23	0.23
Heart rate, bpm	69 ± 26	70 ± 23	0.72
Hypertension, n (%)	38 (59)	33 (50)	0.33
Diabetes, n (%)	33 (51)	31 (47)	0.66
Coronary heart disease, n (%)	31 (48)	35 (53)	0.54
Smoking, n (%)	38 (59)	39 (59)	0.94
Surgery type, n (%)			0.45
Heart surgery	41 (63.1)	46 (69.7)	
General surgery	8 (12.3)	7 (10.6)	
Orthopaedic surgery	2 (3.1)	5 (7.6)	
Urological surgery	8 (12.3)	6 (9.1)	
Vascular surgery	6 (9.2)	2 (3.0)	
Radial artery diameter, mm	2.30 ± 0.50	2.38 ± 0.50	0.36
Median radial artery depth (IQR), mm	3.10 (2.10–4.00)	2.70 (2.10–3.80)	0.40

Abbreviation: *IQR* interquartile range

### Primary outcomes

Regarding the primary outcome, the first-pass success rate without posterior wall puncture was 53.8% in the DNTP group and 44.6% in the AD group (RR = 1.22, 95% CI 0.86–1.72, *P* = 0.26; Table 2).

**Table 2:** Trial results

	Technique		***P*** Value	Relative Risk (95% CI)
	DNTP (***n*** = 65)	AD (***n*** = 66)		
**Primary outcome**				
First-pass success without posterior wall puncture, %	35 (53.8)	29 (44.6)	0.26^a^	1.22 (0.86–1.72)
**Secondary outcomes**				
First-pass success, %	35 (53.8)	39 (59.1)	0.54^a^	0.90 (0.63–1.27)
10-min overall success, %	56 (86.2)	53 (80.3)	0.37^a^	1.22 (0.82–1.81)
Median cannulation time (IQR), s	79.7 (54–109)	47.6 (25–104)	0.007 ^b^	
Posterior wall puncture, %	19 (29.2)	37 (56.1%)	0.002 ^a^	0.56 (0.42–0.82)
Number of skin punctures			0.86 ^a^	
1	50	52		
2	3	1		
≥ 3	12	13		

Abbreviation: *IQR* interquartile range

^a^Chi-square test

^b^Mann-Whitney U test

### Secondary outcomes

Regarding the secondary outcomes, the first-pass success rate was 53.8% in the DNTP group and 59.1% in the AD group (RR = 0.90, 95% CI 0.63–1.27, *P* = 0.54; Table 2). The overall success rate was 86.2% in the DNTP group and 80.3% in the AD group (RR = 1.22, 95% CI 0.82–1.81, *P* = 0.37; Table 2). The time to cannulate the artery was significantly longer in the DNTP group, at 79.65 (54.3–109.4) seconds, than in the AD group, at 47.6 (24.9–103.8) seconds, (*P* = 0.01; Table 2). The posterior wall puncture rate was significantly lower in the DNTP group (29.2%) than in the AD group (56.1%) (RR = 0.56, 95% CI 0.42–0.82, *P* = 0.002; Table 2). There was no significant difference between the two groups in the number of skin punctures (Table 2).

## Discussion

This trial demonstrates that as ultrasound-guided out-of-plane radial artery cannulation techniques, the DNTP and AD techniques are both effective in adult surgical patients. However, although there was no significant difference in the success rate of the two methods, each method has its own advantages and disadvantages. Compared with the AD method, the DNTP method reduced the incidence of posterior wall puncture but also required additional time. In clinical practice, we sometimes encounter patients with poor coagulation and are worried that the arterial puncture will break through the posterior wall and cause complications, such as hematoma. According to our results, it may be more appropriate to use the DNTP technique in these cases. In other cases, such as patients in shock, we are most concerned about catheterizing the artery as soon as possible. In such instances, we may recommend the AD method. Our results could serve as a reference for the above clinical issues.

Recently, many clinical studies have demonstrated the superiority of ultrasound-guided arterial catheterization. Several meta-analyses have shown that ultrasound-guided methods are superior to palpation methods in adults, children, and even newborns [16–19]. According to previous comparisons of out-of-plane ultrasound-guided versus palpation for arterial catheterization, the traditional out-of-plane ultrasound-guided technique, similar to our AD technique, has a first-pass success rate between 53 and 78% [11, 15]. Recently, researchers reported that a modified technique, the DNTP technique, has a first-pass success rate of 82% [6], suggesting that the success rate was higher for the DNTP technique than the traditional AD method. However, there have been no direct comparisons of these two out-of-plane methods. More importantly, ultrasound-guided arterial catheterization has been recommended for its higher success rate and lower complication rate. However, to the best of our knowledge, no published reports have evaluated both the success and complication rates concurrently. Importantly, posterior wall puncture may cause problems, such as hematoma [9]. Therefore, we used the rate of first-pass success without posterior wall puncture as our primary outcome to direct compare these two methods.

We found that there were no significant differences in the rate of first-pass success without posterior wall puncture. This is different from our preliminary trial results, which could be attributed to the following: first, we might have overestimated the difference between the two methods in the preliminary trial; second, the sample size might not have been sufficient. Compared to the results of previously reported studies, in our trial, the first-pass success rates and overall success rates were different for both methods. We think that these differences are more representative of the experience ranges of different operators at each research center than of differences between the methods, as our operator had already conducted DNTP and AD techniques in over 100 patients each before the trial and was equally skilled in the 2 methods.

Ultrasound-guided vascular cannulation includes an in-plane technique and out-of-plane technique [13]. Using in-plane technology, the needle trajectory from needle insertion to catheter placement is clearly visible, which seems to be effective for reducing the incidence of posterior vessel wall puncture [9]. However, this technology requires the operator to be very skilled at ultrasound technology, which is more dependent on experience and can be difficult for novices to master; on the other hand, given that the long axis is subject to slice-thickness artifacts, due to the measurable thickness of the ultrasound beam itself, the cannula in the long axis can sometimes appear to be in the same plane as the extremely small radial artery, even when the cannula has not been successfully inserted into the artery [20, 21]. Therefore, we prefer the out-of-plane technique.

Arterial catheterization consists of two steps: the tip of the needle enters the blood vessel, and the catheter is then placed into the blood vessel [6]. Out-of-plane ultrasound guidance methods can assist in accurately locating the target artery and penetrating the anterior wall of the artery. The subsequent step is similar to that used in traditional palpation. In the DNTP method, the needle tip needs to be observed in the artery lumen, and this may explain why compared to the AD method, the DNTP method significantly reduces the chance of posterior wall perforation. Another concern of using ultrasound is the time required. Our trial shows that compared to the AD method, the DNTP method significantly reduced the probability of penetrating the posterior wall of the artery but also takes more time to complete the procedure. We speculate that the reason for this difference is not only that the DNTP method requires more time for ultrasound probe use but also that the time required is related to the operator’s experience with the ultrasound probe. As the operator gains experience with ultrasound, the difference in the time between the two methods might decrease.

In fact, ultrasound-guided arterial catheterization involves many details and techniques, such as the previously reported “follow the tip” [14] and “saline injection” methods [20]. From this perspective, the DNTP and AD methods are not static but are constantly evolving. In addition, technological developments in ultrasound machines and probes can have a significant impact on the operator, especially the novice. We should pay attention to these details and techniques, continue to improve them, and finally find the ideal, standardized method to achieve the goal of truly benefiting patients with less harm.

There are several limitations to our trial. First, the ultrasound-guided arterial cannulation procedures were performed by a senior resident; therefore, one should be cautious about applying our data to other practitioners. Second, we may have overestimated the difference in our primary outcome between the two methods in the preliminary trial, and if the sample size had been larger, the results might have been different.

In conclusion, there was no significant difference in the rate of first-pass success without posterior wall puncture, the first-pass success rate or the overall success rate between the DNTP and AD methods for ultrasound-guided radial artery cannulation. The DNTP method reduced the incidence of posterior wall puncture of the target artery but required more time. These conclusions could serve as a reference for anesthesiologists when choosing which method to use for arterial cannulation in a specific clinical situation.

## Conclusions

There were no significant differences in the first-pass success rate, with or without arterial posterior wall puncture, or in the 10-min overall success rate between the DNTP and AD groups. However, the cannulation time was longer and the posterior wall puncture rate was lower in the DNTP group than in the AD group. The appropriate technique should be applied depending on the specific clinical situation.

## Data Availability

The datasets used and analyzed during the current study are available from the corresponding author on request.

## Abbreviations

DNTP: Dynamic needle tip positioning
AD: Angle-distance
ASA: American Society of Anesthesiologists
IQR: Interquartile range.
